# Comparable outcomes for TBI-based versus treosulfan based conditioning prior to allogeneic hematopoietic stem cell transplantation in AML and MDS patients

**DOI:** 10.1038/s41409-024-02295-2

**Published:** 2024-05-03

**Authors:** Philipp Berning, Lina Kolloch, Christian Reicherts, Simon Call, Julia Marx, Matthias Floeth, Eva Esseling, Julian Ronnacker, Jörn Albring, Christoph Schliemann, Georg Lenz, Matthias Stelljes

**Affiliations:** 1 Department of Hematology and Oncology, University Hospital Muenster, Muenster, Germany; 2 Center for Molecular and Cellular Oncology, Yale School of Medicine, New Haven, CT, USA

**Keywords:** Acute myeloid leukaemia, Myelodysplastic syndrome

## Abstract

Allogeneic hematopoietic stem cell transplantation (allo-HCT) is a standard treatment for patients with AML and MDS. The combination of fractionated total body irradiation(8GyTBI/Flu) with fludarabine is an established conditioning regimen, but fludarabine/treosulfan(Flu/Treo) constitutes an alternative in older/comorbid patients. We conducted a retrospective analysis of 215 AML(in CR) and 96 MDS patients undergoing their first allo-HCT between 2011 and 2022, identifying 53 matched Flu/Treo and 8GyTBI/Flu patients through propensity score matching. Median follow-up of survivors was 3.3 years and 4.1 years. For the Flu/Treo group, 1-year non-relapse mortality (2% vs. 10%, *p* = 0.03) was lower, while 1-year relapse incidence (16% vs. 13%, *p* = 0.81) was similar. Three-year outcomes, including relapse-free survival and graft-versus-host disease incidence, were comparable (OS: 81% vs. 74%, *p* = 0.70; RFS: 78% vs. 66%, *p* = 0.28; chronic GvHD: 34% vs. 36%, *p* = 0.97; acute GvHD (100 days): 11% vs. 23%, *p* = 0.11). Multivariable analysis, considering age, ECOG, HCT-CI, and MRD status, revealed no associations with main outcomes. Dose-reduced conditioning with Flu/Treo or 8GyTBI/Flu demonstrated favorable and comparable survival rates exceeding 70% at 3 years with 1-year NRM rates below 10% and low relapse rates in the matched cohort. These data underline the need for further evaluation of TBI and Treo-based conditionings in prospective trials.

## Introduction

Although new induction therapies for AML and/or implementation of target therapies have increased response and survival rates of patients with AML or MDS, allogeneic hematopoietic cell transplantation (HCT) remains a valuable curative-intent treatment but carries a significant risk of morbidity and mortality. Due to improvements of supportive care, optimization of transplant procedures and developments of effective treatments for the prevention and treatment of transplant complications, an increasing number of older adults are receiving allogeneic HCT [1]. In many cases, death in remission after transplantation is primarily attributed to infections and graft-versus-host disease (GvHD) but also includes other complications of allo-HCT [2]. To reduce the treatment risk for patients who are not candidates for traditionally used myeloablative conditioning (MAC) regimens and in whom the underlying disease has been well controlled, reduced-intensity conditioning (RIC) or non-myeloablative regimens have been developed [3]. MAC regimens are typically linked to a reduced risk of disease relapse but are accompanied by a higher risk for non-relapse related mortality (NRM). Therefore, RIC regimens are of particular importance for patients aged 60 years or older or those with underlying medical conditions, as they may not be suitable candidates for MAC [4–6].

Fractionated total body irradiation TBI (8 Gy) combined with fludarabine (8GyTBI/Flu) represents an established preparative regimen for AML patients in complete remission (CR). In a randomized study, this regimen showed a significantly lower risk of NRM without corresponding increase in the risk of relapse, compared to a classical full toxic conditioning [7, 8]. More recently, a preparative regimen consisting of treosulfan in combination with fludarabine (Flu/Treo), has emerged as an alternative standard treatment, especially for patients aged ≥ 55 years or those with pre-existing medical conditions. In a large, randomized trial, this regimen has shown significantly higher post-transplant survival rates compared to a long established busulfan-based RIC [9]. To date, there is no comprehensive clinical study that prospectively evaluates the efficacy and toxicity profiles of 8GyTBI/Flu and Flu/Treo. TBI is associated with some, in part, long-term complications due to radiation-related side effects, including endocrine and gonadal disorders, cardiac complications, and lung fibrosis [10, 11]. This may in part explain the lower NRM rates in patients with AML in CR1 aged > 55 years who underwent conditioning with Flu/Treo compared to 8GyTBI/Flu in an analysis of the European Society for Blood and Marrow Transplantation (EBMT) registry [12]. However, this registry-based analysis is limited by data availability and a relatively short follow-up time, leaving unanswered questions regarding the prognostic impact of measurable residual disease, cytogenetic parameters, and comorbidities. Additionally, comparative data on outcomes of both conditioning regimens in patients with myelodysplastic syndromes (MDS) is not available. We aimed to compare outcomes in a large cohort of patients with AML (in CR) and MDS who underwent first allo-HCT to determine the true impact of these regimens on transplant outcomes and to evaluate potentially underlying prognostic factors that might influence clinical decision making with respect to selection of the optimal conditioning strategy.

## Methods

### Data collection

Clinical data were retrospectively extracted from the medical records and electronic patient files. We retrospectively analyzed 311 patients with AML or MDS who received their first allo-HCT after preparatory treatment with either Flu/Treo or 8GyTBI/Flu. Inclusion criteria, treatment modalities and definitions are described in detail in the supplemental material.

### Statistical analysis

All outcomes were calculated from the day of transplantation. Surviving patients were censored at the time of the last contact. We conducted propensity score matching (PSM) in a 1:1 ratio between the Flu/Treo and 8GyTBI/Flu treatment groups. PSM and statistical tests are described in detail in the supplemental material.

## Results

### Patient characteristics and transplant modalities

In total, we identified 311 patients (215 AML and 96 MDS) patients meeting the inclusion criteria, of which 207 patients were treated with Flu/Treo and 104 patients with 8GyTBI/Flu as conditioning therapy. Baseline characteristics grouped by conditioning therapy for the entire cohort are shown in Table 1. Across conditioning therapy groups, we noted significant differences in disease characteristics between the Flu/Treo and 8GyTBI/Flu group in terms of median age at allo-HCT [64 years (range: 19–76) vs. 47 years (range: 18–69)], proportion of AML patients [59.4% vs. 88.5%, *p* < 0.001], ECOG PS [ECOG 2-3: 16.0% vs. 6.8%, *p* = 0.002], proportion of de novo AML [59.3% vs. 87.0%, *p* < 0.001], complex karyotype in cytogenetics [20.5% vs. 9.9%, *p* = 0.023] and adverse/intermediate risk AML (ELN 2017) [77.3% vs. 67.4%, *p* = 0.011]. Further differences were observed with a higher proportion of AML patients with measurable residual disease (available for 196/215 AML patients) in the Flu/Treo group [68.3% vs. 47.8%, *p* = 0.018], more transplantations from HLA-matched-related donors [24.2% vs. 14.4%, *p* = 0.046], higher proportion of HCT-CI Score ≥3 [49.8% vs. 25.0%, *p* < 0.001] and the less frequent application of in vivo T-cell depletion with ATLG (Neovii) [77.3% vs. 87.5%, *p* = 0.033]. Follow-up time was significantly shorter for the Flu/Treo group (2.7 vs. 3.5 years, *p* = 0.003). For GvHD prophylaxis, nearly all cases relied on a combination of cyclosporin A and either MTX or MMF. Supplementary Table 1 additionally displays baseline characteristics grouped by the conditioning group and disease.

**Table 1:** Clinical and transplantation characteristics of all patients by conditioning group before matching.

	Flu/Treo (*N* = 207)	8GyTBI/Flu (*N* = 104)	*P* value
**Diagnosis**			
AML	123 (59.4%)	92 (88.5%)	<0.001
MDS	84 (40.6%)	12 (11.5%)	
**AML Diagnosis Groups**			
De novo AML	73 (59.3%)	80 (87.0%)	<0.001
Secondary AML	37 (30.1%)	10 (10.9%)	
Therapy-related AML	13 (10.6%)	2 (2.2%)	
*MDS patients*	84	12	
**Age at allo-HCT (years)**			
Median [Min, Max]	64.0 [19.0, 76.0]	47.0 [18.0, 69.0]	<0.001
**Sex**			
Female	84 (40.6%)	47 (45.2%)	0.466
Male	123 (59.4%)	57 (54.8%)	
**ECOG score**			
0	8 (3.9%)	13 (12.5%)	0.002
1	166 (80.2%)	84 (80.8%)	
2	32 (15.5%)	6 (5.8%)	
3	1 (0.5%)	1 (1.0%)	
**Cytogenetics: complex karyotype**			
No	163 (79.5%)	91 (90.1%)	0.023
Yes	42 (20.5%)	10 (9.9%)	
Missing	2	3	
**ELN2017 risk classification**			
favorable	28 (22.8%)	30 (32.6%)	0.011
intermediate	43 (35.0%)	41 (44.6%)	
adverse	52 (42.3%)	21 (22.8%)	
*MDS patients*	84	12	
**IPSS-R risk classification**			
low risk	4 (4.8%)	0 (0%)	0.837
intermediate risk	17 (20.2%)	3 (25.0%)	
high risk	63 (75.0%)	9 (75.0%)	
*AML patients*	123	92	
**HCT-CI Score**			
0	52 (25.1%)	45 (43.3%)	<0.001
1-2	52 (25.1%)	33 (31.7%)	
>=3	103 (49.8%)	26 (25.0%)	
**Time-to-transplant in months**			
Median [Min, Max]	3.82 [1.51, 112]	3.93 [0, 98.5]	0.274
**Treosulfan dose**		NA	NA
3 × 10 g/m^2^	206 (99.5%)		
3 × 12 g/m^2^	0		
3 × 14 g/m^2^	1 (0.5%)		
**MRD status pre-transplant**			
MRD negative	30 (24.4%)	38 (41.3%)	0.018
MRD positive	84 (68.3%)	44 (47.8%)	
No Marker	5 (4.1%)	7 (7.6%)	
Missing Information	4 (3.3%)	3 (3.3%)	
*MDS patients*	84	12	
**Donor type**			
Matched-related	50 (24.2%)	15 (14.4%)	0.046
10/10 HLA-matched unrelated	132 (63.8%)	68 (65.4%)	
9/10 HLA-matched unrelated	25 (12.1%)	21 (20.2%)	
**In vivo T-cell depletion**			
No	47 (22.7%)	13 (12.5%)	0.033
Yes	160 (77.3%)	91 (87.5%)	
**Follow-up of survivors in months**			
Median [Min, Max]	32.8 [0.395, 68.0]	42.4 [4.97, 124]	0.003
**GvHD prevention**			
Cyclosporin A + MTX / MMF	205 (99.0%)	104 (100%)	0.553
Tacrolimus + MTX / MMF	2 (1.0%)	0 (0%)	

*AML* acute myelogenous leukemia, *MDS* myelodysplastic neoplasia, *allo-HCT* allogeneic hematopoietic stem cell transplantation, *ECOG* Eastern Cooperative Oncology Group score, *ELN2017* European Leukemia Net 2017 classification, *IPSSR* Revised International Prognostic Scoring System for myelodysplastic syndromes risk assessment, *HCT-CI* hematopoietic cell transplantation-specific comorbidity Index, NA not available, *MRD* measurable residual disease, *HLA* human leukocyte antigens, *GvHD* graft-versus-host disease, *MTX* methotrexate, *MMF* mycophenolate mofetil.

### Key outcomes for unmatched patients

We performed Kaplan-Meier analyses for RFS and OS, as well as cumulative incidences or relapse, NRM and GvHD for the unmatched cohort as summarized in Supplementary Table 2. For the Flu/Treo-cohort, 1-year, and 3-year RFS after allo-HCT was 79% and 63%, respectively, for the 8GyTBI/Flu-patients 81% and 72% (both *p* = 0.240). Overall survival was not significantly different with a 3-year OS of 71% for the Flu/Treo cohort and 79% for the 8GyTBI/Flu cohort (*p* = 0.061)] (Supplementary Fig. 1). We observed higher cumulative incidence of NRM at 1 year for the Flu/Treo group when compared to 8GyTBI/Flu with 8.4% vs. 4.8% (*p* = 0.037) [3-year NRM: 16% vs. 7%], while cumulative relapse incidence at 1-year and 3-years were similar across both groups [1-year: 13% vs. 14%; 3-year: 21% vs. 21%; *p* = 0.750] (Supplementary Fig. 2). In terms of GvHD incidences, no significant differences for acute GvHD grade II-IV (*p* = 0.294), grade III-IV (*p* = 0.454) and chronic GvHD (*p* = 0.054) were noted (Supplementary Fig. 3).

### Propensity score matching

To address the substantial differences observed in key outcomes and clinical parameters with known prognostic significance between patients treated with Flu/Treo and 8GyTBI/Flu, we conducted propensity score matching (PSM) using age at allo-HCT, sex, underlying disease (AML or MDS) as matching parameters. The baseline characteristics of the matched patient cohort are presented in Table 2. A total of 106 matched patients were grouped based on the conditioning therapy (PSM-Flu/Treo-cohort, PSM-8GyTBI/Flu-cohort). As presented in Table 2, baseline characteristics were balanced for PSM-Flu/Treo and PSM-8GyTBI/Flu patients with respect to age (57 vs. 55 years, *p* = 0.203), female patients (49.1% vs. 45.3%, *p* = 0.846), underlying disease (AML: 83.0% vs. 83.0%, *p* = 1.000), and HCT-CI score groups (HCT-CI ≥ 3: 41.5% vs. 39.6%, *p* = 0.179). Of note, the Flu/Treo group showed a significantly higher rate of ECOG scores of 2-3 compared to the comparison group (17.0% vs. 7.6%, *p* = 0.014). Further disease characteristics were equally distributed. In particular, the proportion of patients with de novo AML was comparable in the PSM-Flu/Treo and PSM-8GyTBI/Flu group [72.7 vs. 79.5%, *p* = 0.618], complex karyotype was found in 11.3% vs. 9.8% (*p* = 1.000) and no significant differences in the distribution ELN2017 risk categories for AML (adverse risk: 40.9% vs. 31.8%, *p* = 0.637) as well as IPSS-R risk groups for MDS (high/very high risk: 88.9% vs. 66.7%, *p* = 0.576) emerged. The distribution of transplant characteristics revealed consistent proportions across several factors, including MRD status prior to allo-HCT for AML patients (*p* = 1.000), donor types (*p* = 0.144) and the utilization of in vivo T-cell depletion (*p* = 0.092) as shown in Table 2. Nevertheless, it’s noteworthy that the PSM-Flu/Treo cohort exhibited a significantly shorter median time from diagnosis to allo-HCT [3.6 months (range: 1.9–58.9) vs. 4.3 months (range: 1.9–39.7), *p* = 0.046]. Furthermore, Supplementary Table 3 provides an overview of the clinical characteristics within the PSM cohort, by conditioning regimen and underlying disease. Among all 106 PSM-matched patients, AML patients were older than MDS patients at allo-HCT [median: 56 (27–71) vs. 47 years (19–65)], while the distribution of other clinical characteristics was comparable for both disease groups.

**Table 2:** Clinical and transplantation characteristics by conditioning groups for matched patients.

	Flu/Treo (*N* = 53)	8GyTBI/Flu (*N* = 53)	*P* value
**Diagnosis**			
AML	44 (83.0%)	44 (83.0%)	1.000
MDS	9 (17.0%)	9 (17.0%)	
**AML Diagnosis Groups**			
De novo AML	32 (72.7%)	35 (79.5%)	0.618
Secondary AML	12 (27.3%)	9 (20.5%)	
MDS	9	9	
**Age at allo-HCT (years)**			
Median [Min, Max]	57.0 [19.0, 71.0]	55.0 [27.0, 69.0]	0.203
**Sex**			
Female	26 (49.1%)	24 (45.3%)	0.846
Male	27 (50.9%)	29 (54.7%)	
**ECOG score**			
0	0 (0%)	5 (9.4%)	0.014
1	44 (83.0%)	44 (83.0%)	
2	9 (17.0%)	3 (5.7%)	
3	0 (0%)	1 (1.9%)	
**Cytogenetics: Complex karyotype**			
No	47 (88.7%)	46 (90.2%)	1.000
Yes	6 (11.3%)	5 (9.8%)	
Missing	0	2	
**ELN2017 classification**			
favorable	10 (22.7%)	13 (29.5%)	0.637
intermediate	16 (36.4%)	17 (38.6%)	
adverse	18 (40.9%)	14 (31.8%)	
*MDS patients*	9	9	
**IPSS-R**			
low risk	0 (0%)	0 (0%)	0.576
intermediate risk	1 (11.1%)	3 (33.3%)	
high/very high risk	8 (88.9%)	6 (66.7%)	
*AML patients*	44	44	
**HCT-CI Score**			
0	17 (32.1%)	10 (18.9%)	0.179
1-2	14 (26.4%)	22 (41.5%)	
>=3	22 (41.5%)	21 (39.6%)	
**Time-to-transplant in months**			
Median [Min, Max]	3.59 [2.04, 58.9]	4.34 [1.91, 39.7]	0.046
**Treosulfan dose**		NA	NA
3 × 10 g/m^2^	52 (98.1%)		
3 × 12 g/m^2^	0		
3 × 14 g/m^2^	1 (1.9%)		
**MRD status at allo-HCT**			
MRD negative	15 (34.1%)	15 (34.1%)	1.000
MRD positive	24 (54.5%)	24 (54.5%)	
Not evaluated	3 (6.8%)	3 (6.8%)	
Missing Information	2 (4.5%)	2 (4.5%)	
*MDS patients*	9	9	
**Donor type**			
Matched-related	17 (32.1%)	8 (15.1%)	0.144
10/10 HLA-matched unrelated	28 (52.8%)	35 (66.0%)	
9/10 HLA-matched unrelated	8 (15.1%)	10 (18.9%)	
**In vivo T-cell depletion**			
No	15 (28.3%)	7 (13.2%)	0.092
Yes	38 (71.7%)	46 (86.8%)	
**Median follow-up of survivors (months)**			
Median [Min, Max]	40.0 [6.25, 64.7]	49.7 [4.97, 124]	0.084
**GvHD prevention**			
Cyclosporin A + MTX / MMF	52 (98.1%)	53 (100%)	1.000
Tacrolimus + MTX / MMF	1 (1.9%)	0 (0%)	

*AML* acute myelogenous leukemia, *MDS* myelodysplastic neoplasia, *allo-HCT* allogeneic hematopoietic stem cell transplantation, *ECOG* Eastern Cooperative Oncology Group score, *ELN2017* Euorpean LeukemiaNet 2017 classification, *IPSSR* Revised International Prognostic Scoring System for myelodysplastic syndromes risk assessment, *HCT-CI* hematopoietic cell transplantation-specific comorbidity Index, *NA* not available, *MRD* measurable residual disease, *HLA* human leukocyte antigens, *GvHD* graft-versus-host disease, *MTX* methotrexate, *MMF* mycophenolate mofetil.

### Key outcomes for matched patients

**Fig. 1:**
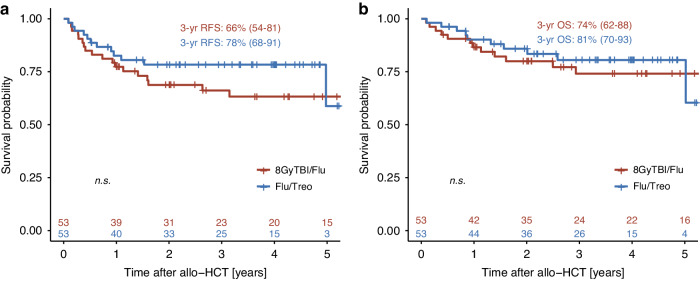
Kaplan-Meier estimates for matched patients by conditioning groups. Relapse-free survival (**a**) and overall survival (**b**) for propensity score matched (PSM) patients with Flu/Treo and 8GyTBI/Flu conditioning.

**Fig. 2:**
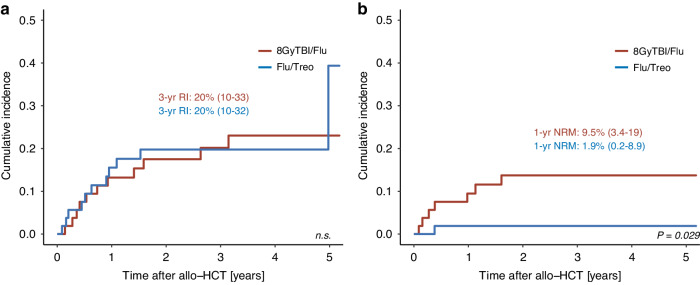
Cumulative incidences of relapse and NRM for matched patients by conditioning groups. Cumulative incidences of relapse (**a**) and non-relapse mortality (**b**) for propensity score matched (PSM) patients with Flu/Treo and 8GyTBI/Flu conditioning.

With a median follow-up of alive patients of 3.3 years in the Flu/Treo and 4.1 years in the 8GyTBI/Flu group (*p* = 0.084), Kaplan-Meier estimates for RFS were similar (Fig. 1, Table 3). In the PSM-Flu/Treo-cohort, 83%, 78% and 59% patients showed a RFS at 1, 3 and 5 years after allo-HCT, respectively, opposed to 77%, 66% and 63% (*p* = 0.283) in the PSM-8GyTBI/Flu-cohort (Table 3). One and 3-year OS were comparable in the PSM-Flu/Treo and 8GyTBI/Flu groups with 90% vs. 87% and 81% vs. 74% (both *p* = 0.704), respectively (Table 3). There were no statistically significant differences between Flu/Treo and 8GyTBI/Flu in terms of cumulative relapse incidence (3 year: 20% vs. 20%, *p* = 0.811), while 1-year NRM (1.9% vs. 9.5%, *p* = 0.029) was lower for the Flu/Treo group (Table 3, Fig. 2). Among matched patients aged ≥ 55 years, NRM was found to be higher for those who underwent 8GyTBI/Flu conditioning, with a 1-year NRM of 14%, while no NRM event occurred for the Flu/Treo-treated patients (*p* = 0.015). No significant differences were noted between both conditioning regimens for patients ≥55 years of age with respect to OS (*p* = 0.253), RFS (*p* = 0.179) and relapse incidence (*p* = 0.897). Furthermore, the propensity score matching model was additionally employed for AML patients to account for potential differences between the two diseases. For 40 AML pair-matched patients, 3-year RFS and OS rates in the Flu/Treo group was 71% and 76%, respectively, compared to 66% (*p* = 0.929) and 76% (*p* = 0.436) in the and 8GyTBI/Flu group. There were no significant differences for 1-year NRM (5.1% vs. 7.5%, *p* = 0.722) and relapse (3-year: 20% vs. 27%, *p* = 0.940).

**Table 3:** Univariate outcomes by conditioning groups.

	Flu/Treo	8 GyTBI/Flu	
Outcomes	Probability (95% CI)	Probability (95% CI)	*P* value
**Acute GvHD Grade II-IV**			0.112^a^
100 days	11% (4.6%, 22%)	23% (12%, 35%)	
6 months	17% (8.3%, 28%)	32% (20%, 45%)	
**Acute GvHD Grade III-IV**			0.749^a^
100 days	5.7% (1.5%, 14%)	5.7% (1.5%, 14%)	
6 months	7.5% (2.4%, 17%)	9.4% (3.4%, 19%)	
**Chronic GvHD**			0.979^a^
1 year	27% (16%, 40%)	19% (9.6%, 30%)	
3 years	34% (21%, 47%)	36% (23%, 50%)	
5 years	37% (23%, 50%)	39% (25%, 52%)	
**Non-relapse mortality**			0.029^a^
1 year	1.9% (0.15%, 8.9%)	9.5% (3.4%, 19%)	
3 years	1.9% (0.15%, 8.9%)	14% (5.9%, 25%)	
5 years	1.9% (0.15%, 8.9%)	14% (5.9%, 25%)	
**Relapse Incidence**			0.811^a^
1 year	16% (7.2%, 27%)	13% (5.7%, 24%)	
3 years	20% (10%, 32%)	20% (10%, 33%)	
5 years	39% (6.6%, 73%)	23% (12%, 36%)	
**Relapse-free survival**			0.283
1 year	83% (73%, 94%)	77% (67%, 89%)	
3 years	78% (68%, 91%)	66% (54%, 81%)	
5 years	59% (33%, 100%)	63% (51%, 79%)	
**Overall survival**			0.704
1 year	90% (82%, 99%)	87% (78%, 96%)	
3 years	81% (70%, 93%)	74% (62%, 88%)	
5 years	81% (70%, 93%)	74% (62%, 88%)	

^a^Gray’s test.

*Flu/Treo* fludarabine/treosulfan, *8* *GyTBI/Flu* 8Gy total body irradiation/fludarabine, *CI* confidence interval, *GvHD* graft-versus-host disease.

**Fig. 3:**
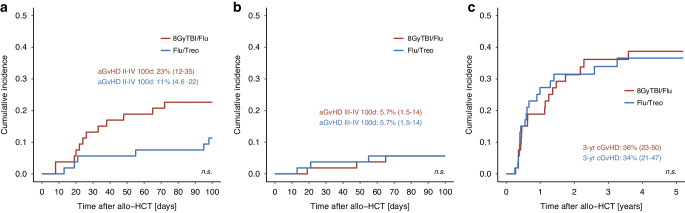
Cumulative incidences of acute and chronic GvHD for matched patients by conditioning groups. Cumulative incidences of acute GvHD Grade II-IV (**a**), acute GvHD III-IV (**b**), and chronic GvHD (**c**) for propensity score matched (PSM) patients with Flu/Treo and 8GyTBI/Flu conditioning. GvHD Graft-versus-host disease.

Infection was the leading cause of NRM in the PSM-8GyTBI/Flu group (4/7 patients) and PSM-Flu/Treo-group (1/1 patients). Two PSM-8GyTBI/Flu patients died from GvHD-related causes, and one patient deceased due to mesenteric ischemia. Importantly, no secondary malignancies were observed in the PSM cohort. Additionally, no significant differences between PSM-Flu/Treo and PSM-8GyTBI/Flu in the cumulative incidence of acute GvHD II-IV at day 100 (11% vs. 23%, *p* = 0.112), acute GvHD III–IV at day 100 (5.7% vs. 5.7%, *p* = 0.749) or chronic GvHD at 3 years (34% vs. 36%, *p* = 0.979) were observed (Fig. 3). Next, we performed univariable and multivariable Cox regression analysis for RFS and OS as shown in Table 4. After adjusting for conditioning groups, age group ( > 60 years), HCT-CI score groups and MRD status, we did not observe any underlying factors associated with the key outcomes. Similarly, we also employed the Cox regression model in the unmatched cohort; however, we were unable to identify factors associated with the key outcomes in the multivariate model (Supplementary Table 4).

**Table 4:** Univariate and multivariate Cox proportional hazards models for RFS and OS.

		Relapse-free Survival		Overall Survival	
Variable	Category	HR (univariable)	HR (multivariable)	HR (univariable)	HR (multivariable)
Treatment Group	8GyTBI/Flu	–	–	–	–
	Flu/Treo	0.67 (0.32–1.40, *p* = 0.286)	0.74 (0.28–1.97, *p* = 0.542)	0.85 (0.36–1.98, *p* = 0.704)	1.55 (0.45–5.37, *p* = 0.486)
Diagnosis	AML	–	–	–	–
	MDS	1.66 (0.74–3.72, *p* = 0.215)	–	1.72 (0.68–4.37, *p* = 0.253)	–
AML Diagnosis Groups	De novo AML	–	–	–	–
	Secondary AML	1.40 (0.58–3.42, *p* = 0.456)	–	0.97 (0.31–2.99, *p* = 0.959)	–
Age at allo-HCT (years) (continuous variable)	Mean (SD)	1.01 (0.97–1.06, *p* = 0.515)	1.03 (0.97–1.10, *p* = 0.347)	1.02 (0.97–1.08, *p* = 0.363)	1.05 (0.97–1.13, *p* = 0.268)
Age >60 years	No	–	–	–	–
	Yes	1.82 (0.81–4.09, *p* = 0.146)	–	1.76 (0.69–4.49, *p* = 0.236)	–
Sex	Female	–	–	–	–
	Male	0.86 (0.42–1.75, *p* = 0.680)	–	0.74 (0.32–1.70, *p* = 0.473)	–
ECOG score	0–1	–	–	–	–
	2–3	1.49 (0.57–3.92, *p* = 0.415)	1.39 (0.42–4.65, *p* = 0.588)	2.34 (0.86–6.39, *p* = 0.098)	1.89 (0.52–6.85, *p* = 0.336)
Cytogenetics: complex karyotype	No	–	–	–	–
	Yes	2.49 (1.01–6.14, *p* = 0.047)	–	2.76 (1.02–7.50, *p* = 0.046)	–
ELN2017 risk classification	Adverse	–	–	–	–
	Favorable	0.59 (0.18–1.93, *p* = 0.388)	–	0.66 (0.20–2.20, *p* = 0.500)	–
	Intermediate	1.16 (0.47–2.86, *p* = 0.754)	–	0.62 (0.20–1.90, *p* = 0.400)	–
HCT-CI Score	0	–	–	–	–
	1–2	1.74 (0.54–5.68, *p* = 0.355)	1.10 (0.25–4.84, *p* = 0.904)	6.35 (0.79–50.90, *p* = 0.082)	3.29 (0.33–32.39, *p* = 0.307)
	≥3	3.07 (1.04–9.09, *p* = 0.042)	2.34 (0.57–9.58, *p* = 0.237)	9.56 (1.26–72.72, p = 0.029)	4.93 (0.51–47.25, *p* = 0.167)
Time Diagnosis-to-Transplant (continuous variable)	Mean (SD)	1.00 (1.00–1.00, *p* = 0.577)	-	1.00 (1.00–1.00, *p* = 0.565)	-
MRD status before allo-HCT	MRD negative	–	–	–	–
	MRD positive	0.98 (0.40–2.41, *p* = 0.969)	0.89 (0.35–2.24, *p* = 0.806)	1.22 (0.41–3.68, *p* = 0.721)	0.99 (0.31–3.14, *p* = 0.980)
Donor type	10/10 HLA-matched unrelated	–	–	–	–
	9/10 HLA-matched unrelated	0.67 (0.25–1.83, *p* = 0.435)	–	1.07 (0.37–3.12, *p* = 0.895)	–
	Matched-related	0.63 (0.24–1.67, *p* = 0.352)	–	1.09 (0.39–3.08, *p* = 0.868)	–
In vivo T-cell depletion	No	–	–	–	–
	Yes	2.37 (0.72–7.84, *p* = 0.157)	–	1.56 (0.46–5.31, *p* = 0.474)	–

*HR* hazard ratio, *8GyTBI/Flu* 8Gy total body irradiation/fludarabine, *Flu/Treo* fludarabine/treosulfan, *AML* acute myelogenous leukemia, *MDS* myelodysplastic neoplasia, *allo-HCT* allogeneic hematopoietic stem cell transplantation, *ECOG* Eastern Cooperative Oncology Group score, *ELN2017* Euorpean LeukemiaNet 2017 classification, *HCT-CI* hematopoietic cell transplantation-specific comorbidity Index, *MRD* measurable residual disease, *HLA* huocyte antigens, *GvHD* graft-versus-host disease, *MTX* methotrexate, *MMF* mycophenolate mofetil.

## Discussion

In our retrospective analysis patients treated with treosulfan-based or 8 Gy TBI-based conditioning prior allogeneic HCT showed no significant differences in survival outcomes or cumulative incidences of relapse or acute / chronic GvHD. Recognizing the need to address potential biases resulting from variations in patient characteristics, we utilized a propensity score matching approach, which enabled us to balance for baseline factors, such as age and HCT-CI scores. Moreover, given the pivotal roles of measurable residual disease (MRD) in AML patient outcomes [13–15], our propensity score matching model successfully addressed these factors. With a sufficient follow-up of 4 years and comprehensive data for disease and patient characteristics, we noted comparable efficacy of both conditioning regimens in all subgroups. In summary, our study results suggest that both regimens, Flu/Treo, and 8GyTBI/Flu, are safe and highly effective conditioning therapies prior allogeneic HCT for patients with AML and MDS.

So far, prospective randomized studies comparing TBI- and non-TBI based conditioning regimens for adult AML / MDS patients are lacking. Traditionally, TBI-based conditioning regimens for patients with AML have been associated with a survival benefits compared to chemotherapy-only conditioning [4, 8]. With the introduction of intravenous busulfan patient outcomes improved and recent large retrospective studies showed no conclusive results [16, 17]. However, most of these studies compared TBI (mainly 12 Gy TBI, given in various fractions) and busulfan based myeloablative conditioning. For AML patients aged 18–60 years a randomized study revealed that, compared to 12GyTBI/cyclophosphamide, 8GyTBI/Flu is associated with a reduced incidence of NRM, which was particularly evident for individuals between 41 and 60 years of age, without resulting in a higher incidence of relapse [8]. Formally, 8GyTBI-based conditioning is not classified as a reduced intensity conditioning (RIC) regimen. However, this reduced-toxicity conditioning (RTC) regimen has shown to be feasible in elderly patients and / or patients not eligible for MAC [7]. Of note, the transplant conditioning intensity (TCI) weighted risk score categorizes the applied Flu/Treo regimen (virtually all patients had a treosulfan dose of 30 g/m^2^) as a low intensity regimen with a score of 1.5, while the applied 8 Gy TBI/Flu regimen is positioned within the intermediate risk TCI category with a score of 2.5 [18]. A register study from the EBMT compared 8GyTBI based RTC with busulfan based MAC and showed better overall survival and reduced relapse incidences particular for patients aged up to 50 years, whereas for patients aged 50 years or older the use of 8GyTBI/Flu was associated with increased incidence of NRM [19]. However, the reported NRM rate of 26% at 2 years observed in patients ≥50 years was relatively high and might also reflect different strategies in supportive care, donor selection and levels of center experience [19].

Within the multicenter MC-FludT.14/L trial, 570 AML and MDS patients aged 50–70 years or <50 years with a comorbidity index (HCT-CI) of >2, were randomized to 30 g/m^2^ treosulfan or 6.4 mg/kg of busulfan [9]. Both agents were combined with standard dosages of fludarabine. The estimated EFS and OS at 3 years was significantly better for patients receiving treosulfan compared to busulfan based conditioning (60% vs. 50% and 67% vs. 56%, respectively). While 3-year relapse incidences (26%) were identical between both groups, the cumulative incidence for non-relapse related mortality was significantly lower for the treosulfan group (14% vs. 21%) [9]. Our comparative cohort study revealed that both, 8 Gy TBI and treosulfan based conditioning might allow relevant reduction of disease relapse. While the efficacy of 8GyTBI/Flu was comparable to the results reported from Bug et al., the relapse incidence for the Flu/Treo cohort was significantly higher with 35% at 2 years [12]. Of note, the NRM rate of 28% in elderly patients was significantly higher after TBI-based conditioning in this EBMT study and might in part be explained by difference in the fractions of TBI applied and radiation technique (not reported for the EBMT-cohort) [12]. We believe this finding might be in part attributed to a standardized TBI delivery approach employed at our center utilizing a PRIMUS linear accelerator or a TrueBeam linear accelerator. This methodology is generally associated with relatively low toxicity rates as previously shown [20]. The observed overall toxicity, represented by NRM rates for both conditioning regimens, was generally comparable to major randomized trials [4, 7–9]. For Flu/Treo, our findings suggested a potentially more favorable NRM risk compared to the pivotal trial, which could be attributed to differences in age distributions (median age at allo-HCT 56 years vs. 60 years) [9]. Additionally, the large EBMT analysis by Nagler et al. could show comparable NRM results for CR1 AML patients with 8.5% at 5 years (median age 57 years) [21]. On the other hand, the 1-year-NRM rates with <10% for 8GyTBI/Flu were in line with those reported in the randomized trial [8, 22].

Interestingly, the cumulative incidence of chronic GvHD in our matched cohort appeared more favorable when compared to a randomized trial, likely due to a relatively high proportion of patients receiving in vivo T-cell depletion [9]. Regarding measurable residual disease at transplantation for AML patients, our study comprehensively assessed the molecular or cytogenetic aberrations detectable at allo-HCT. This single-center approach allowed for a more consistent evaluation in contrast to registry-based multicenter analyses which may suffer from variations and limited data availability in MRD assessment [12, 21, 23]. While many reports highlight the prognostic relevance of MRD status prior to allo-HCT, we could not identify MRD positivity as an independent prognostic factor in our matched cohort [14, 23]. In line with this finding, the EBMT analysis by Bug et al. failed to retrospectively show a prognostic relevance of MRD status for these conditioning regimens in CR1 AML patients, despite the registry-related limitations of MRD evaluation [12]. However, it has been shown in a large cohort that RIC conditioning for AML (in CR) and MDS in patients with detectable molecular alterations evaluated by a comprehensive NGS panel, was associated with an increased risk of relapse and decreased outcomes as opposed to MAC-conditioning [6]. Due to the real-world nature of our data and potential inconsistencies in data availability or the depth of molecular analysis, these findings necessitate further research in a larger, more comprehensive setting that also encompasses a wider range of RIC protocols. Notably, we did not identify any prognostic parameters, including AML or MDS risk groups and adverse cytogenetics, that significantly influenced survival outcomes. These results were almost identical to those reported from studies that used busulfan-, TBI- and treosulfan-based reduced intensity conditioning regimens [8, 9, 12, 22]. The primary cause of treatment failure remained relapse, consistent with the results of randomized trials for both conditioning regimens [8, 9]. Our findings, along with data from Bug et al. and the phase 3 trial, suggest higher NRM in patients ≥ 55 years receiving TBI conditioning indicating that treosulfan-based conditioning is effective should be the preferred option [9, 12]. However, further investigation through well-designed clinical trials is warranted. These trials could directly compare TBI-based conditioning to treosulfan-conditioning and exploring different treosulfan doses within treosulfan-based regimens as an effective alternative for younger patients.

The present study has limitations. While we employed robust propensity score matching techniques, unknown factors may still have influenced the outcomes. Additionally, the single-center design potentially limits the generalizability of our findings to other populations or clinical settings. The relatively small sample size constrained our ability to detect significant differences between the two regimens. While propensity score matching (PSM) effectively reduced confounding variables between our initially different patient groups, the resulting relatively small number of patients in each group may limit the ability to detect statistically significant differences between the TBI-based conditioning and non-TBI conditioning arms. While we cannot completely rule out the inclusion of outcome data from a subset of AML patients in an EBMT registry analysis, which may affect less than 20% of our total cohort, our analysis exceeds such registry-based analyses in terms of data depth. Nevertheless, our study, with its long follow-up of more than 4 years and comprehensive data availability, contributes valuable insights into the outcomes of AML and MDS patients undergoing allogeneic HCT with Flu/Treo or 8GyTBI/Flu conditioning.

Our data underline the effectiveness of both Flu/Treo and 8GyTBI/Flu conditioning regimens for patients with AML in CR or MDS, each demonstrating an acceptable toxicity profile. Particularly, treosulfan-based conditioning showed a low NRM rate and comparable efficacy when compared to TBI-based conditioning. Detectable MRD for AML patients did not constitute a prognostic factor in our study. The results of our study form the basis for future prospective studies comparing TBI and non-TBI-based reduced-toxicity conditioning regimen with an additional focus on disease status prior transplantation, radiation technique and supportive care, particularly GvHD prophylaxis.

### Supplementary information

Supplemental Material

## Data Availability

The original contributions presented in the study are included in the article/Supplementary material. Further inquiries can be directed to the corresponding author.
