# Supplementary material for: Comparable outcomes for TBI-based versus treosulfan based conditioning prior to allogeneic hematopoietic stem cell transplantation in AML and MDS patients
Source: Bone Marrow Transplant. 2024 May 3;59(8):1097–106. doi: 10.1038/s41409-024-02295-2 (PMC11296947; doi:10.1038/s41409-024-02295-2)
Supplement: Supplementary file 1 — Supplemental Material [file 41409_2024_2295_MOESM1_ESM.docx]

**Supplementary Information**

**Comparable outcomes for TBI-based versus treosulfan based conditioning prior to allogeneic hematopoietic stem cell transplantation in AML and MDS patients**

Philipp Berning^1,2*^, Lina Kolloch^1*^, Christian Reicherts^1^, Simon Call^1^, Julia Marx^1^, Matthias Floeth^1^, Eva Esseling^1^, Julian Ronnacker^1^, Jörn Albring^1^, Christoph Schliemann^1^, Georg Lenz^1^, Matthias Stelljes^1^

^1^ Department of Hematology and Oncology, University Hospital Muenster, Muenster, Germany

^2^ Center for Molecular and Cellular Oncology, Yale School of Medicine, New Haven, USA

^*^ Contributed equally to this work as co-first authors.

**Correspondence:** Matthias Stelljes, Department of Hematology and Oncology, University Hospital Münster, Münster, Germany, Phone: +49 251 8352801, Email: stelljes@uni-muenster.de.

**Supplemental Methods**

## *Data collection*

## In our analysis the following inclusion criteria were applied: diagnosis of AML in complete hematologic remission (CR) at allo-HCT or MDS; first allo-HCT from a 10/10 HLA-matched related donor, 10/10 or 9/10 HLA-matched unrelated donor; peripheral blood stem cells or bone marrow as stem cell source; preparatory treatment with either fludarabine (any dose) and treosulfan (dose of 30 or 42 g/m^2^) [Flu/Treo] or fludarabine (any dose) and fractionated TBI 8 Gy (4×2Gy) [8GyTBI/Flu] with following allo-HCT at the University Hospital Muenster, Germany, between April 2011 and October 2022.

TBI for the majority was delivered using a PRIMUS or TrueBeam linear accelerator allowing for precise doses of radiation in a small number of fractions as outlined previously ^1^. In brief, this technique is routinely used to reduce potential toxicities due to sharp dose gradients outside the target volume, limiting the dose to nearby organs and side effects. In vivo dosimetry was utilized across eight measurement positions. For the maximum 8 Gy dosage administered in our cohort, no additional mediastinal shielding was required. Outcome data from a portion of our cohort may have been incorporated into EBMT analysis by But et al., which includes allo-HCT for AML patients from 2009 to 2019. This could impact 60 out of 311 patients (19.3%) or 60 out of 215 AML patients (27.9%), respectively, who also met the inclusion criteria used in the EBMT analysis ^2^.

The study was approved by the local ethics committee (2022-645-f-S), and all patients signed informed consent for retrospective analyses prior to transplantation. All study procedures were performed in accordance with relevant guidelines, such as the Declaration of Helsinki, as well as local regulations.

*Definitions*

The primary endpoints analyzed in this study were overall survival (OS) and relapse-free survival (RFS) calculated from the day of allo-HCT. OS was defined as survival until death from any cause. RFS was defined as survival without relapse after allo-HCT (CR; defined as <5% blasts in the bone marrow). Secondary endpoints were relapse incidence (RI) and non-relapse mortality (NRM). Disease relapse was defined as blast count ≥5% in post-transplantation bone marrow analysis by cytology or flow cytometry and/or the presence of extramedullary involvement confirmed through histopathological analysis of the respective manifestation. NRM was defined as death without previous AML or MDS relapse and/or progression. AML patients were retrospectively risk-stratified according to the European Leukemia Net (ELN) 2017 classification system ^3^. Detectable measurable residual disease (MRD) in AML patients was defined as any detectable molecular/cytogenetic alteration prior to allo-HCT. Cytogenetic risk classification for MDS patients was evaluated retrospectively according to the revised International Prognostic Scoring System (IPSS-R) scoring system ^4^. Definitions of complex karyotype were applied according to international criteria ^3, 4^.

*Statistical Analysis*

All outcomes were calculated from the day of transplantation. Surviving patients were censored at the time of the last contact. The probabilities of RFS and OS were calculated using the Kaplan–Meier method. We calculated cumulative incidences for relapse and NRM using a competing risk model, where death during remission was treated as a competing event for relapse. Death and relapse were considered as competing events for calculations of acute GvHD and chronic GvHD. Clinical characteristics were compared between Flu/Treo- and 8GyTBI/Flu-treated patients using the chi-squared test or Fisher’s exact test for categorical variables and the Mann–Whitney U test for continuous variables.

Propensity score matching was based on nearest-neighbor matching, considering the following co-variates: underlying disease (AML or MDS), age at the time of allo-HCT, sex and Hematopoietic Cell Transplantation-specific Comorbidity Index (HCT-CI) score. To ensure robust matching, we applied a caliper of 0.3 standard deviations of propensity scores. For a more comprehensive understanding of the PSM methodology, additional details can be retrieved elsewhere ^5^. In our PSM analysis, we compared a total of 53 patients in each treatment group.

Univariable analyses were performed using the log-rank test for RFS and OS and Gray’s test was used for cumulative incidences. Multivariable analyses for RFS and OS were performed using the Cox proportional-hazards regression model. The results were presented as hazard ratios (HR) with the corresponding 95% confidence intervals (95% CI). All statistical tests were two-sided, and we maintained a fixed Type I error level of 0.05 for factors associated with time-to-event outcomes. All statistical analyses were conducted using R statistical software version 4.2.2, accessible online at http://www.R-project.org.

**Supplemental Figures**

## Supplemental Figure 1. Kaplan-Meier estimates for all patients by conditioning groups.

## Relapse-free survival (A) and overall survival (B) for propensity score matched (PSM) patients with Flu/Treo and 8GyTBI/Flu conditioning.

## Supplemental Figure 2. Cumulative incidences of relapse and NRM for all patients by conditioning groups.

## Cumulative incidences of relapse (A) and non-relapse mortality (B) for propensity score matched (PSM) patients with Flu/Treo and 8GyTBI/Flu conditioning.

## Supplemental Figure 3. Cumulative incidences of acute and chronic GvHD for all patients by conditioning groups.

## Cumulative incidences of acute GvHD Grade II-IV (A), acute GvHD III-IV (B), and chronic GvHD (C) for propensity score matched (PSM) patients with Flu/Treo and 8GyTBI/Flu conditioning. GvHD=Graft-versus-host disease.

## Supplemental Figure 1. Kaplan-Meier estimates for all patients by conditioning groups.

**
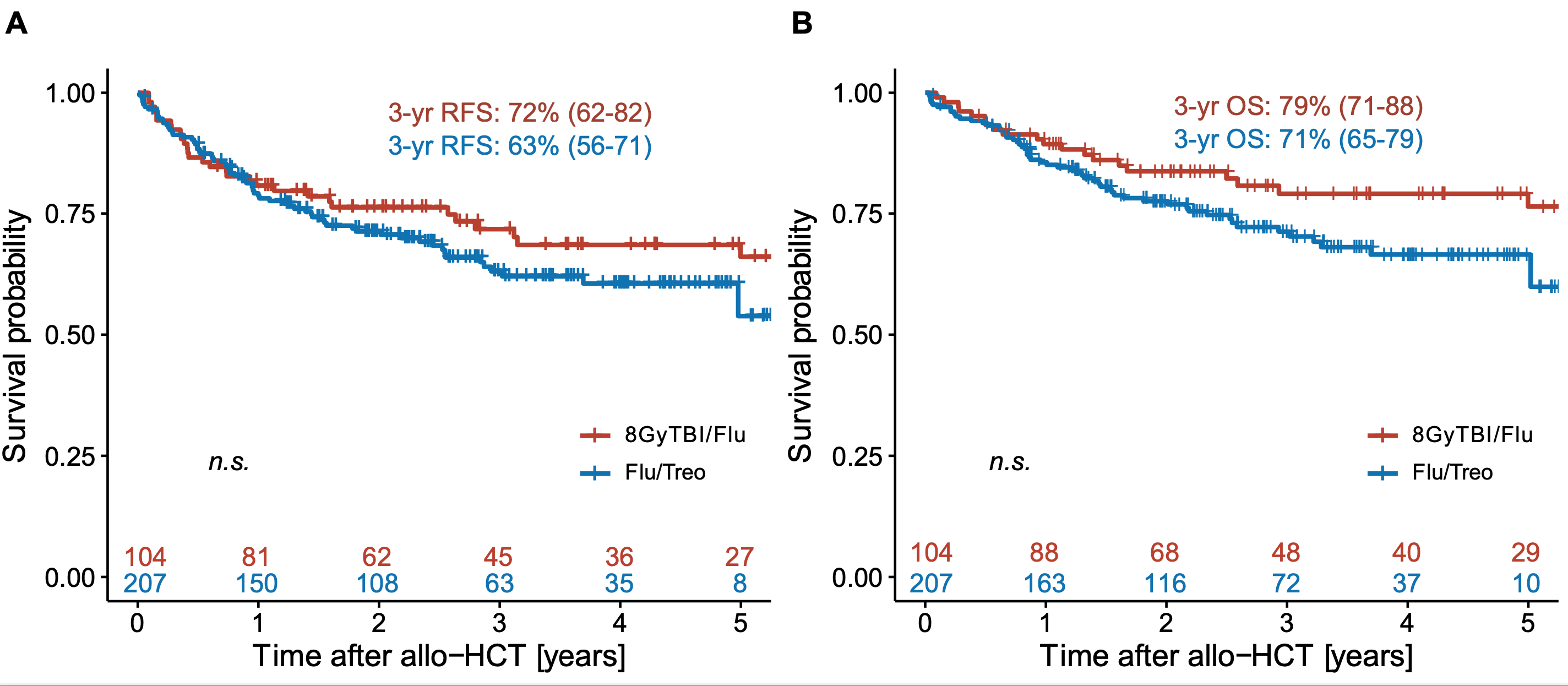
**

## Supplemental Figure 2. Cumulative incidences of relapse and NRM for all patients by conditioning groups.


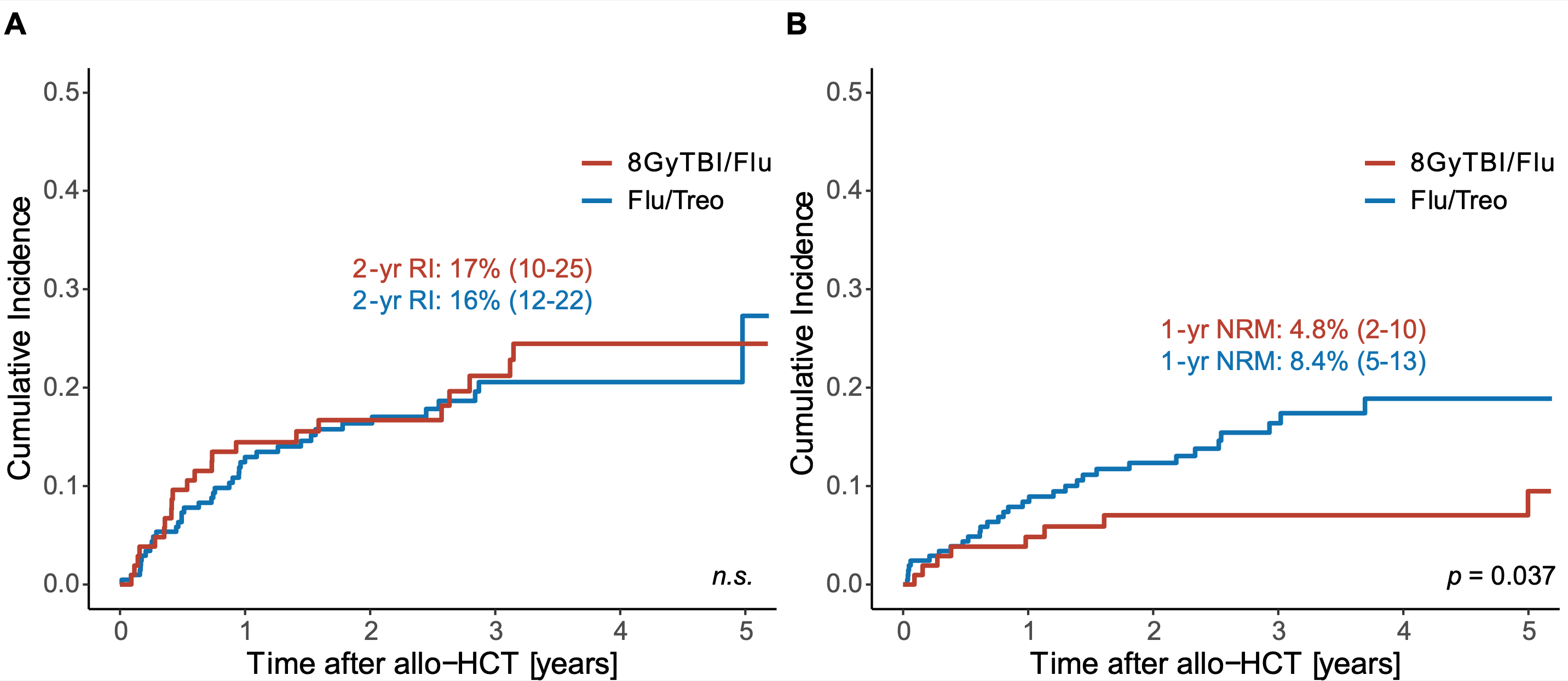


## Supplemental Figure 3. Cumulative incidences of acute and chronic GvHD for all patients by conditioning groups.


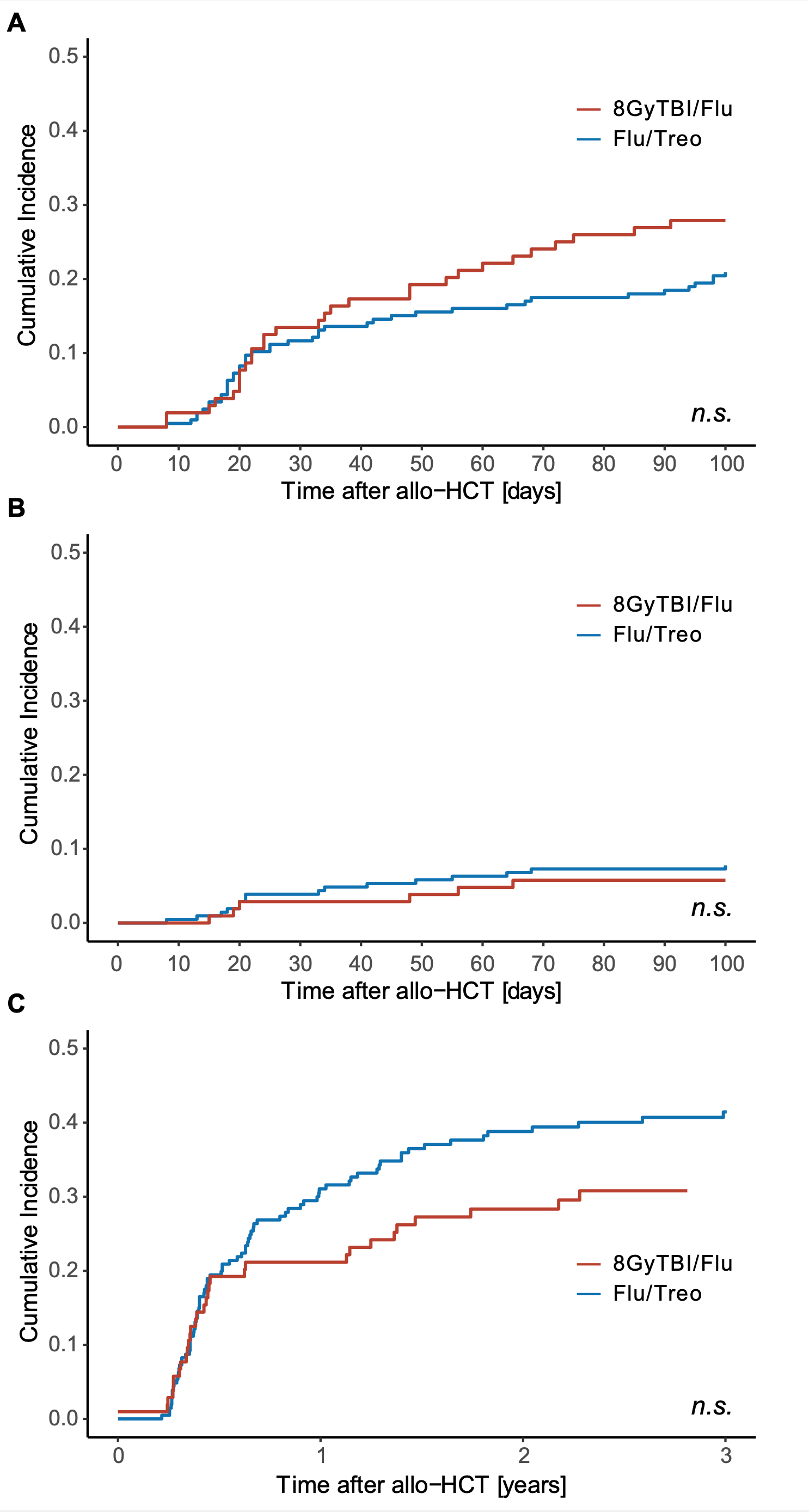


## Supplemental Tables

**Supplemental Table 1. Baseline characteristics of all patients by conditioning groups and disease before matching.**

|  | **Total** | | **Flu/Treo** | | **8GyTBI/Flu** | |
| --- | --- | --- | --- | --- | --- | --- |
|  | **AML (N=215)** | **MDS (N=96)** | **AML (N=123)** | **MDS (N=84)** | **AML (N=92)** | **MDS (N=12)** |
| **Age at allo-HSCT (years)** |  |  |  |  |  |  |
| Median [Min, Max] | 58.0 [18.0, 75.0] | 62.0 [19.0, 76.0] | 64.0 [32.0, 75.0] | 63.0 [19.0, 76.0] | 48.5 [18.0, 69.0] | 46.5 [22.0, 65.0] |
| **Sex** |  |  |  |  |  |  |
| Female | 98 (45.6%) | 33 (34.4%) | 54 (43.9%) | 30 (35.7%) | 44 (47.8%) | 3 (25.0%) |
| Male | 117 (54.4%) | 63 (65.6%) | 69 (56.1%) | 54 (64.3%) | 48 (52.2%) | 9 (75.0%) |
| **ECOG score** |  |  |  |  |  |  |
| 0 | 14 (6.5%) | 7 (7.3%) | 3 (2.4%) | 5 (6.0%) | 11 (12.0%) | 2 (16.7%) |
| 1 | 172 (80.0%) | 78 (81.3%) | 96 (78.0%) | 70 (83.3%) | 76 (82.6%) | 8 (66.7%) |
| 2 | 27 (12.6%) | 11 (11.5%) | 23 (18.7%) | 9 (10.7%) | 4 (4.3%) | 2 (16.7%) |
| 3 | 2 (0.9%) | 0 (0%) | 1 (0.8%) | 0 (0%) | 1 (1.1%) | 0 (0%) |
| **AML Diagnosis Groups** |  |  |  |  |  |  |
| De novo AML | 153 (71.2%) |  | 73 (59.3%) |  | 80 (87.0%) |  |
| Secondary AML | 47 (21.9%) |  | 37 (30.1%) |  | 10 (10.9%) |  |
| Therapy-related AML | 15 (7.0%) |  | 13 (10.6%) |  | 2 (2.2%) |  |
| MDS patients | 0 | 96 | 0 | 84 | 0 | 12 |
| **Cytogenetics: complex karyotype** |  |  |  |  |  |  |
| No | 190 (90.5%) | 64 (66.7%) | 109 (90.1%) | 54 (64.3%) | 81 (91.0%) | 10 (83.3%) |
| Yes | 20 (9.5%) | 32 (33.3%) | 12 (9.9%) | 30 (35.7%) | 8 (9.0%) | 2 (16.7%) |
| Missing | 5 | 0 | 2 | 0 | 3 | 0 |
| **ELN2017 risk classification** |  |  |  |  |  |  |
| favorable | 58 (27.0%) |  | 28 (22.8%) |  | 30 (32.6%) |  |
| intermediate | 84 (39.1%) |  | 43 (35.0%) |  | 41 (44.6%) |  |
| adverse | 73 (34.0%) |  | 52 (42.3%) |  | 21 (22.8%) |  |
| MDS patients | 0 | 96 | 0 | 84 | 0 | 12 |
| **IPSS-R risk classification** |  |  |  |  |  |  |
| low risk |  | 4 (4.2%) |  | 4 (4.8%) |  | 0 (0%) |
| intermediate risk |  | 20 (20.8%) |  | 17 (20.2%) |  | 3 (25.0%) |
| high risk |  | 72 (75.0%) |  | 63 (75.0%) |  | 9 (75.0%) |
| AML patients | 215 | 0 | 123 | 0 | 92 | 0 |
| **HCT-CI Score** |  |  |  |  |  |  |
| 0 | 68 (31.6%) | 29 (30.2%) | 27 (22.0%) | 25 (29.8%) | 41 (44.6%) | 4 (33.3%) |
| 1-2 | 60 (27.9%) | 25 (26.0%) | 30 (24.4%) | 22 (26.2%) | 30 (32.6%) | 3 (25.0%) |
| >=3 | 87 (40.5%) | 42 (43.8%) | 66 (53.7%) | 37 (44.0%) | 21 (22.8%) | 5 (41.7%) |
| **Median Time-to-transplant in months** |  |  |  |  |  |  |
| Median [Min, Max] | 3.75 [0, 98.5] | 4.39 [1.91, 112] | 3.49 [1.51, 86.1] | 4.65 [1.94, 112] | 3.93 [0, 98.5] | 4.06 [1.91, 6.64] |
| **MRD status pre-transplant** |  |  |  |  |  |  |
| MRD negative | 68 (31.6%) |  | 30 (24.4%) |  | 38 (41.3%) |  |
| MRD positive | 128 (59.5%) |  | 84 (68.3%) |  | 44 (47.8%) |  |
| No Marker | 12 (5.6%) |  | 5 (4.1%) |  | 7 (7.6%) |  |
| Missing Information | 7 (3.3%) |  | 4 (3.3%) |  | 3 (3.3%) |  |
| *MDS patients* | 0 | 96 | 0 | 84 | 0 | 12 |
| **Donor type** |  |  |  |  |  |  |
| Matched-related | 48 (22.3%) | 17 (17.7%) | 34 (27.6%) | 16 (19.0%) | 14 (15.2%) | 1 (8.3%) |
| 10/10 HLA-matched unrelated | 139 (64.7%) | 61 (63.5%) | 77 (62.6%) | 55 (65.5%) | 62 (67.4%) | 6 (50.0%) |
| 9/10 HLA-matched unrelated | 28 (13.0%) | 18 (18.8%) | 12 (9.8%) | 13 (15.5%) | 16 (17.4%) | 5 (41.7%) |
| **In vivo T-cell depletion** |  |  |  |  |  |  |
| No | 44 (20.5%) | 16 (16.7%) | 31 (25.2%) | 16 (19.0%) | 13 (14.1%) | 0 (0%) |
| Yes | 171 (79.5%) | 80 (83.3%) | 92 (74.8%) | 68 (81.0%) | 79 (85.9%) | 12 (100%) |
| **Median follow-up of survivors in months** |  |  |  |  |  |  |
| Median [Min, Max] | 35.2 [0.395, 117] | 33.4 [5.76, 124] | 32.8 [0.395, 62.7] | 32.2 [5.76, 68.0] | 40.3 [4.97, 117] | 57.1 [12.9, 124] |
| **GvHD prevention** |  |  |  |  |  |  |
| Cyclosporin A + MTX / MMF | 214 (99.5%) | 95 (99.0%) | 122 (99.2%) | 83 (98.8%) | 92 (100%) | 12 (100%) |
| Tacrolimus + MTX / MMF | 1 (0.5%) | 1 (1.0%) | 1 (0.8%) | 1 (1.2%) | 0 (0%) | 0 (0%) |

AML=acute myelogenous leukemia; MDS=myelodysplastic neoplasia; allo-HCT=allogeneic hematopoietic stem cell transplantation; ECOG= Eastern Cooperative Oncology Group score; ELN2017=Euorpean LeukemiaNet 2017 classification; IPSSR=Revised International Prognostic Scoring System for myelodysplastic syndromes risk assessment; HCT-CI=hematopoietic cell transplantation-specific comorbidity Index; MRD=measurable residual disease; HLA=human leukocyte antigens; GvHD=graft-versus-host disease; MTX=methotrexate; MMF=mycophenolate mofetil.

**Supplemental Table 2. Univariate Outcomes by conditioning groups before matching.**

|  | **Flu/Treo**  **(N=207)** | **8GyTBI/Flu**  **(N=104)** |  |
| --- | --- | --- | --- |
| **Outcomes** | **Probability (95% CI)** | **Probability (95% CI)** | **P-value** |
| **Acute GvHD Grade II-IV** |  |  | 0.294* |
| 100 days | 21% (16%, 27%) | 28% (20%, 37%) |  |
| 6 months | 26% (20%, 32%) | 36% (26%, 45%) |  |
| **Acute GvHD Grade III-IV** |  |  | 0.454* |
| 100 days | 7.8% (4.6%, 12%) | 5.8% (2.4%, 11%) |  |
| 6 months | 9.2% (5.8%, 14%) | 8.7% (4.2%, 15%) |  |
| **Chronic GvHD** |  |  | 0.054* |
| 1 year | 31% (25%, 38%) | 21% (14%, 29%) |  |
| 2 years | 39% (32%, 46%) | 28% (20%, 37%) |  |
| 3 years | 41% (34%, 48%) | 31% (22%, 40%) |  |
| **Non-relapse mortality** |  |  | 0.037 * |
| 1 year | 8.4% (5.1%, 13%) | 4.8% (1.8%, 10%) |  |
| 2 years | 12% (8.2%, 17%) | 7% (3.1%, 13%) |  |
| 3 years | 16% (11%, 22%) | 7% (3.1%, 13%) |  |
| **Relapse Incidence** |  |  | 0.750* |
| 1 year | 13% (8.7%, 18%) | 14% (8.5%, 22%) |  |
| 2 years | 16% (12%, 22%) | 17% (10%, 25%) |  |
| 3 years | 21% (15%, 27%) | 21% (13%, 30%) |  |
| **Relapse-free survival** |  |  | 0.240 |
| 1 year | 79% (73%, 85%) | 81% (73%, 89%) |  |
| 2 years | 71% (65%, 78%) | 76% (68%, 85%) |  |
| 3 years | 63% (56%, 71%) | 72% (63%, 82%) |  |
| **Overall survival** |  |  | 0.061 |
| 1 year | 86% (81%, 91%) | 89% (84%, 95%) |  |
| 2 years | 78% (72%, 84%) | 84% (77%, 91%) |  |
| 3 years | 71% (65%, 79%) | 79% (71%, 88%) |  |
| **^*^** Gray’s test  Flu/Treo=fludarabine/treosulfan; 8GyTBI/Flu=8Gy total body irradiation/fludarabine; CI=confidence interal;  GvHD=graft-versus-host disease. | | | |

## Supplemental Table 3. Clinical and transplantation characteristics by conditioning groups and disease after matching.

|  | **Total** | | **Flu/Treo** | | **8GyTBI/Flu** | |
| --- | --- | --- | --- | --- | --- | --- |
|  | **AML (N=88)** | **MDS (N=18)** | **AML (N=44)** | **MDS (N=9)** | **AML (N=44)** | **MDS (N=9)** |
| **Age at allo-HSCT (years)** |  |  |  |  |  |  |
| Median [Min, Max] | 56.0 [27.0, 71.0] | 47.0 [19.0, 65.0] | 58.0 [32.0, 71.0] | 42.0 [19.0, 59.0] | 55.0 [27.0, 69.0] | 47.0 [45.0, 65.0] |
| **Sex** |  |  |  |  |  |  |
| Female | 42 (47.7%) | 8 (44.4%) | 21 (47.7%) | 5 (55.6%) | 21 (47.7%) | 3 (33.3%) |
| Male | 46 (52.3%) | 10 (55.6%) | 23 (52.3%) | 4 (44.4%) | 23 (52.3%) | 6 (66.7%) |
| **ECOG score** |  |  |  |  |  |  |
| 0 | 3 (3.4%) | 2 (11.1%) | 0 (0%) | 0 (0%) | 3 (6.8%) | 2 (22.2%) |
| 1 | 72 (81.8%) | 16 (88.9%) | 35 (79.5%) | 9 (100%) | 37 (84.1%) | 7 (77.8%) |
| 2 | 12 (13.6%) | 0 (0%) | 9 (20.5%) | 0 (0%) | 3 (6.8%) | 0 (0%) |
| 3 | 1 (1.1%) | 0 (0%) | 0 (0%) | 0 (0%) | 1 (2.3%) | 0 (0%) |
| **Cytogenetics: Complex karyotype** |  |  |  |  |  |  |
| No | 80 (93.0%) | 13 (72.2%) | 41 (93.2%) | 6 (66.7%) | 39 (92.9%) | 7 (77.8%) |
| Yes | 6 (7.0%) | 5 (27.8%) | 3 (6.8%) | 3 (33.3%) | 3 (7.1%) | 2 (22.2%) |
| Missing | 2 | 0 | 0 | 0 | 2 | 0 |
| **ELN2017 classification** |  |  |  |  |  |  |
| favorable | 23 (26.1%) | NA | 10 (22.7%) | NA | 13 (29.5%) | NA |
| intermediate | 33 (37.5%) | NA | 16 (36.4%) | NA | 17 (38.6%) | NA |
| adverse | 32 (36.4%) | NA | 18 (40.9%) | NA | 14 (31.8%) | NA |
| *MDS patients* | 0 | 18 | 0 | 9 | 0 | 9 |
| **IPSS-R** |  |  |  |  |  |  |
| low risk | NA | 0 (0%) | NA | 0 (0%) | NA | 0 (0%) |
| intermediate risk | NA | 4 (22.2%) | NA | 1 (11.1%) | NA | 3 (33.3%) |
| high risk | NA | 14 (77.8%) | NA | 8 (88.9%) | NA | 6 (66.7%) |
| *AML patients* | 88 | 0 | 44 | 0 | 44 | 0 |
| **HCT-CI score** |  |  |  |  |  |  |
| 0 | 21 (23.9%) | 6 (33.3%) | 13 (29.5%) | 4 (44.4%) | 8 (18.2%) | 2 (22.2%) |
| 1-2 | 31 (35.2%) | 5 (27.8%) | 12 (27.3%) | 2 (22.2%) | 19 (43.2%) | 3 (33.3%) |
| >=3 | 36 (40.9%) | 7 (38.9%) | 19 (43.2%) | 3 (33.3%) | 17 (38.6%) | 4 (44.4%) |
| Missing |  |  |  |  |  |  |
| **Median Time-to-transplant in months** | 3.85 [1.94, 58.9] | 3.96 [1.91, 9.93] | 3.44 [2.04, 58.9] | 3.88 [2.73, 9.93] | 4.72 [1.94, 39.7] | 4.21 [1.91, 6.64] |
| Median [Min, Max] |  |  |  |  |  |  |
| **MRD status pre-transplant** | 30 (34.1%) | NA | 15 (34.1%) | NA | 15 (34.1%) | NA |
| MRD negative | 48 (54.5%) | NA | 24 (54.5%) | NA | 24 (54.5%) | NA |
| MRD positive | 6 (6.8%) | NA | 3 (6.8%) | NA | 3 (6.8%) | NA |
| No Marker | 4 (4.5%) | NA | 2 (4.5%) | NA | 2 (4.5%) | NA |
| Not evaluated | 0 | 18 | 0 | 9 | 0 | 9 |
| *MDS patients* |  |  |  |  |  |  |
| **Donor type** | 25 (28.4%) | 0 (0%) | 17 (38.6%) | 0 (0%) | 8 (18.2%) | 0 (0%) |
| Matched-related | 51 (58.0%) | 12 (66.7%) | 22 (50.0%) | 6 (66.7%) | 29 (65.9%) | 6 (66.7%) |
| 10/10 HLA-matched unrelated | 12 (13.6%) | 6 (33.3%) | 5 (11.4%) | 3 (33.3%) | 7 (15.9%) | 3 (33.3%) |
| 9/10 HLA-matched unrelated |  |  |  |  |  |  |
| **In vivo T-cell depletion** | 22 (25.0%) | 0 (0%) | 15 (34.1%) | 0 (0%) | 7 (15.9%) | 0 (0%) |
| No | 66 (75.0%) | 18 (100%) | 29 (65.9%) | 9 (100%) | 37 (84.1%) | 9 (100%) |
| Yes |  |  |  |  |  |  |
| **Median follow-up of survivors in months** | 40.3 [4.97, 112] | 56.9 [10.1, 124] | 40.0 [6.25, 62.7] | 40.0 [10.1, 64.7] | 43.6 [4.97, 112] | 75.5 [49.2, 124] |
| Median [Min, Max] | 17 | 6 | 8 | 2 | 9 | 4 |
| **GvHD prevention** |  |  |  |  |  |  |
| Cyclosporin A + MTX / MMF | 87 (98.9%) | 18 (100%) | 43 (97.7%) | 9 (100%) | 44 (100%) | 9 (100%) |
| Tacrolimus + MTX / MMF | 1 (1.1%) | 0 (0%) | 1 (2.3%) | 0 (0%) | 0 (0%) | 0 (0%) |

AML=acute myelogenous leukemia; MDS=myelodysplastic neoplasia; allo-HCT=allogeneic hematopoietic stem cell transplantation; ECOG= Eastern Cooperative Oncology Group score; ELN2017=Euorpean LeukemiaNet 2017 classification; IPSSR=Revised International Prognostic Scoring System for myelodysplastic syndromes risk assessment; HCT-CI=hematopoietic cell transplantation-specific comorbidity Index; MRD=measurable residual disease; HLA=human leukocyte antigens; GvHD=graft-versus-host disease; MTX=methotrexate; MMF=mycophenolate mofetil.

## Supplemental Table 4. Univariate and multivariate Cox proportional hazards models for RFS and OS before matching.

|  |  | **Relapse-free Survival** | | | **Overall Survival** | | |
| --- | --- | --- | --- | --- | --- | --- | --- |
| **Variable** | **Category** | **HR (univariable)** | **HR (multivariable)** | | **HR (univariable)** | | **HR (multivariable)** |
| Treatment Group | FluTreo | - | - | - | | - | |
|  | FluTBI | 0.77 (0.50-1.19, p=0.241) | 1.06 (0.54-2.10, p=0.860) | 0.61 (0.37-1.03, p=0.064) | | 0.82 (0.37-1.81, p=0.631) | |
| Diagnosis | AML | - | - | - | | - | |
|  | MDS | 1.38 (0.92-2.06, p=0.124) | - | 1.45 (0.91-2.30, p=0.116) | | - | |
| AML Diagnosis Groups | De novo AML | - | - | - | | - | |
|  | Secondary AML | 1.70 (1.03-2.81, p=0.039) | - | 1.68 (0.95-2.99, p=0.077) | | - | |
| Age at allo-HCT (years)  (continuous variable) | Mean (SD) | 1.01 (0.99-1.03, p=0.183) | 1.00 (0.98-1.03, p=0.759) | 1.02 (1.00-1.04, p=0.025) | | 1.01 (0.98-1.04, p=0.570) | |
| Age >60 years | No | - | - | - | | - | |
|  | Yes | 1.28 (0.87-1.90, p=0.213) | - | 1.60 (1.02-2.51, p=0.041) | | - | |
| Sex | Female | - | - | - | | - | |
|  | Male | 0.96 (0.65-1.42, p=0.828) | - | 1.15 (0.73-1.81, p=0.552) | | - | |
| ECOG score | 0-1 | - | - | - | | - | |
|  | 2-3 | 1.86 (1.13-3.09, p=0.016) | 1.70 (0.88-3.27, p=0.113) | 2.37 (1.39-4.04, p=0.001) | | 2.07 (1.02-4.18, p=0.043) | |
| Cytogenetics: complex karyotype | No | - | - | - | | - | |
|  | Yes | 2.33 (1.49-3.64, p<0.001) | - | 2.91 (1.79-4.73, p<0.001) | | - | |
| ELN2017 risk classification | favorable | - | - | - | | - | |
|  | intermediate | 1.95 (0.97-3.93, p=0.062) | - | 1.73 (0.78-3.82, p=0.177) | | - | |
|  | adverse | 2.11 (1.03-4.30, p=0.040) | - | 2.16 (0.98-4.77, p=0.056) | | - | |
| HCT-CI Score | 0 | - | - | - | | - | |
|  | 1-2 | 1.49 (0.86-2.58, p=0.153) | 1.28 (0.63-2.61, p=0.490) | 1.35 (0.72-2.54, p=0.345) | | 1.28 (0.57-2.88, p=0.543) | |
|  | >=3 | 1.75 (1.06-2.87, p=0.027) | 1.36 (0.68-2.71, p=0.379) | 1.81 (1.03-3.16, p=0.038) | | 1.22 (0.56-2.66, p=0.612) | |
| Time Diagnosis-to-Transplant  (continuous variable) | Mean (SD) | 1.00 (1.00-1.00, p=0.638) | - | 1.00 (1.00-1.00, p=0.698) | | - | |
| MRD status before allo-HCT | MRD negative | - | - | - | | - | |
|  | MRD positive | 1.10 (0.64-1.89, p=0.730) | 0.93 (0.52-1.66, p=0.795) | 1.31 (0.69-2.50, p=0.410) | | 0.99 (0.50-1.96, p=0.985) | |
| Donor type | Matched-related | - | - | - | | - | |
|  | 10/10 HLA-matched unrelated | 0.97 (0.59-1.58, p=0.892) | - | 0.83 (0.48-1.45, p=0.521) | | - | |
|  | 9/10 HLA-matched unrelated | 0.97 (0.50-1.88, p=0.931) | - | 0.98 (0.48-2.02, p=0.962) | | - | |
| In vivo T-cell depletion | No | - | - | - | | - | |
|  | Yes | 1.10 (0.66-1.83, p=0.726) | - | 1.03 (0.58-1.84, p=0.921) | | - | |

HR=hazard ratio; 8GyTBI/Flu=8Gy total body irradiation/fludarabine; Flu/Treo=fludarabine/treosulfan; AML=acute myelogenous leukemia; MDS=myelodysplastic neoplasia; allo-HCT=allogeneic hematopoietic stem cell transplantation; ECOG= Eastern Cooperative Oncology Group score; ELN2017=Euorpean LeukemiaNet 2017 classification; HCT-CI=hematopoietic cell transplantation-specific comorbidity Index; MRD=measurable residual disease; HLA=human leukocyte antigens; GvHD=graft-versus-host disease; MTX=methotrexate; MMF=mycophenolate mofeti

References

1. Oertel M, Kittel C, Martel J, Mikesch JH, Glashoerster M, Stelljes M *et al.* Pulmonary Toxicity after Total Body Irradiation-An Underrated Complication? Estimation of Risk via Normal Tissue Complication Probability Calculations and Correlation with Clinical Data. *Cancers (Basel)* 2021; **13**(12). e-pub ahead of print 20210612; doi: 10.3390/cancers13122946

2. Bug G, Labopin M, Niittyvuopio R, Stelljes M, Reinhardt HC, Hilgendorf I *et al.* Fludarabine/TBI 8 Gy versus fludarabine/treosulfan conditioning in patients with AML in first complete remission: a study from the Acute Leukemia Working Party of the EBMT. *Bone Marrow Transplant* 2023; **58**(6)**:** 710-716. e-pub ahead of print 20230331; doi: 10.1038/s41409-023-01965-x

3. Dohner H, Estey E, Grimwade D, Amadori S, Appelbaum FR, Buchner T *et al.* Diagnosis and management of AML in adults: 2017 ELN recommendations from an international expert panel. *Blood* 2017; **129**(4)**:** 424-447. e-pub ahead of print 20161128; doi: 10.1182/blood-2016-08-733196

4. Greenberg PL, Tuechler H, Schanz J, Sanz G, Garcia-Manero G, Sole F *et al.* Revised international prognostic scoring system for myelodysplastic syndromes. *Blood* 2012; **120**(12)**:** 2454-2465. e-pub ahead of print 20120627; doi: 10.1182/blood-2012-03-420489

5. Thoemmes FJ, Kim ES. A Systematic Review of Propensity Score Methods in the Social Sciences. *Multivariate Behav Res* 2011; **46**(1)**:** 90-118. doi: 10.1080/00273171.2011.540475
